# Bystander Program Effectiveness to Reduce Violence Acceptance: RCT in High Schools

**DOI:** 10.1007/s10896-018-9961-8

**Published:** 2018-04-02

**Authors:** Ann L. Coker, Heather M. Bush, Candace J. Brancato, Emily R. Clear, Eileen A. Recktenwald

**Affiliations:** 1 College of Medicine, Department of Obstetrics & Gynecology, University of Kentucky, 800 Rose Street, Lexington, KY 40536 USA; 2 College of Public Health, University of Kentucky, Lexington, KY USA; 3 Kentucky Association of Sexual Assault Programs, 83 C Michael Davenport Blvd, Frankfort, KY 40604 USA

**Keywords:** Bystanding, Violence acceptance, High schools, Sexual violence

## Abstract

Bystander-based violence prevention interventions have shown efficacy to reduce dating violence and sexual violence acceptance at the individual level yet no large randomized controlled trial (RCT) has evaluated this effect at the high-school level and over time. This rigorous cluster-randomized controlled trial addresses this gap by evaluating intervention effectiveness at both school and individual levels. Kentucky high schools were randomized to intervention or control conditions. In intervention schools educators provided school-wide ‘Green Dot’ presentations and bystander training with student popular opinion leaders. Each spring from 2010 to 2014; 73,044 students completed anonymous surveys with no missing data on relevant outcomes. Dating violence and sexual violence acceptance were the primary outcomes for this analysis. At the school level, slopes from linear mixed models using averaged school-level dating violence acceptance (condition–time, *p* < 0.001) and sexual violence acceptance (condition–time interaction, *p* < 0.001) differed indicating a significant reduction in the violence acceptance in the intervention relative to control schools over time and specifically in years 3 and 4 when ‘Green Dot’ was fully implemented. Analyses based on student’s self-reported receipt of ‘Green Dot’ training by condition confirmed the school level finding of significant reductions in both dating violence and sexual violence acceptance in years 3 and 4 for both males and females. In this RCT we find evidence that the bystander-based violence prevention intervention ‘Green Dot’ works, as hypothesized and as implemented, to reduce acceptance of dating violence and sexual violence at the school and individual levels.

## Introduction

Social norms of sexual and dating violence acceptance have been identified as strong correlates of individuals using sexual or dating violence (Banyard 2008; Burn 2009; McMahon 2010). The influence of social norms which condone, support, or excuse sexual and dating violence has received significant attention recently in social, political, and criminal justice settings. (DeGue et al. 2014). Greater acceptance of rape myths or other measures of violence acceptance have been associated with individuals being less likely to use bystander *helping* actions (Banyard 2008; Banyard and Moynihan 2011; McMahon 2010). Those who reported higher self-efficacy were more likely to use engaged bystander behaviors and to have fewer barriers to safely intervene (Banyard et al. 2007; Banyard 2008; Banyard and Moynihan 2011; Palmer et al. 2016; Yule and Grych 2017). Bystander-based violence programming makes use of the connection between violence acceptance and violence rates by creating an intervention with the specific objective of reducing violence by motivating or engaging potential bystanders to act to reduce violence risk. Programming seeks to change bystander actions by first changing the violence acceptance in the trained individual who then influences those within his or her social network. Bystander intervention programs share a philosophy that all members of the community have a role in preventing violence. By engaging participants not as potential victims or perpetrators but as potential allies, both defensiveness and victim-blaming attitudes are reduced (Banyard et al. 2004; Berkowitz 2002; Moynihan and Banyard 2008). The purpose of this study is to evaluate the effectiveness and efficacy of the Green Dot bystander intervention to reduce dating violence and sexual violence acceptance. Because violence acceptance scores differed by sex at baseline (Cook-Craig et al. 2014), analyses were conducted by sex. Analyses of violence acceptance and bystander behaviors were identified in clinicaltrials.gov as secondary or intermediate outcomes. The primary outcomes were sexual violence perpetration and victimization (Coker et al. 2017).

Bystander approaches have been recognized as promising prevention strategies for violence prevention (http://changingourcampus.org). This approach teaches individuals how to recognize situations or behaviors that may become violent and to intervene to reduce the likelihood of violence (Banyard et al. 2004). At the individual level, bystander interventions may reduce violent behaviors by increasing willingness and self-efficacy to challenge violence-supportive norms and behaviors in one’s peer group (Coker et al. 2011). These individual interventions within peer groups can diffuse the benefits of training through social networks to produce changes in social norms and behavior at the community level. Emerging evidence suggests that bystander approaches to violence prevention may increase bystander intentions (Banyard et al. 2007; Brown et al. 2014; Moynihan et al. 2010; Potter 2016); promote positive bystander behaviors (Coker et al. 2011); and reduce violence among college students (Coker et al. 2015, 2016; Gidycz et al. 2011), adolescent male athletes (Miller et al. 2013), and high-school students (Coker et al. 2017).

The current analyses add to the existing research from bystander-based evaluations in our use of a school-based cluster randomized controlled trial (RCT) to evaluate both the effectiveness and efficacy of the intervention on 2 secondary outcomes: dating violence and sexual violence acceptance. Because school-based interventions were hypothesized to have effects at both school, (e.g., changing a school’s culture of support for violence acceptance) and individual student, (e.g., attitudes toward violence) levels, comprehensive analyses at both levels were needed to better understand whether and how an intervention changed hypothesized outcomes. Our analysis is a unique contribution to the existing literature because both school and student level analyses were conducted; the bystander training effect was measured based on school-level randomization (effectiveness measured prospectively) and student level training received (efficacy measured cross-sectionally within year). Conducting both effectiveness and efficacy analyses allows a comprehensive assessment of the impact of this large ‘real world’ bystander intervention on changing the relevant social norm, violence acceptance. The timing between intervention implementation and ability to measure effect had to be considered, because changes at the individual level may occur earlier than changes at a school level. Measuring the effect of training only among those trained would limit the picture of program effectiveness particularly, if modeling behaviors were more effective than training itself. The importance of measuring both school- and student-level effects of training on changes in social norms (here violence acceptance) may be more imperative than that for other outcomes because changes in norms may precede changes in actions (bystander behaviors) and changes in effect (violence). Further, changes in norms can be diffused rapidly within social network through peer pressures.

While bystander interventions have been hypothesized to reduce violence by both reducing violence acceptance and increasing bystander actions, the current analysis focused exclusively on the effect of this intervention on changing violence acceptance.

### Hypothesis 1

In this RCT, in which schools were randomized to either the intervention or control condition, dating and sexual violence acceptance levels (school-level averages) were hypothesized to decline at a greater rate over time in schools randomized to the intervention relative to control schools. (Effectiveness analyses, Intervention as randomized at school level).

### Hypothesis 2

Compared with students attending control schools (no intervention training at the school level), those in intervention schools receiving training by phase would with training, have lower sexual dating violence acceptance scores. (Efficacy analyses, Intervention as received at student level). Phase 1 training was fully implemented in Y1-Y2 and Phase 2 by Y3-Y4.

## Methods

The methods of this RCT have been described in greater detail elsewhere (Coker et al. 2017) and documented in https://clinicaltrials.gov/show/NCT01878097. Data collection began in 2010; no data analyses were conducted before final data collection and cleaning in late 2014 (Coker et al. 2017). The primary outcomes for the RCT were sexual violence perpetration and victimization. Secondary or intermediate outcomes were intervention-associated changes in a violence acceptance and bystander behaviors. The present analyses focuses on intervention-associated changes in the two measures violence acceptance.

Briefly, the Green Dot bystander-based sexual violence prevention program was selected as the intervention to test for this RCT (http://www.alteristic.org) by the Prevention Intervention Committee of the Kentucky Association of Sexual Assault Programs. Green Dot’s purpose was to change violence acceptance of trained students and to engage students as potential bystanders to safely and effectively act to reduce the risk of interpersonal violence within their social network or community (Coker et al. 2011). Green Dot is theory-based and supported by research drawing from bystander psychology (Chekroun and Brauer 2002; Clark and Word 1974; Latané and Darley 1970) diffusion of innovation theory (Darley and Latané 1968; Rushton and Campbell 1977), and sexual violence perpetrator characteristics (Johnson et al. 2006). Through Green Dot bystander training, male and female students are taught to recognize situations and behaviors that could lead to violence or abuse (termed “red dots”). Students are then trained to identify active bystander behaviors they can take either individually or collectively (called “green dots”) to reduce the risk or effect of violence. Green Dot can be distinguished from other bystander-based intervention (e.g., Bringing in the Bystander, MVP, “Coaching Boys into Men”) in its inclusion of the males and females in the same training groups and its use of a popular opinion leader identification and invitation into intervention training model (Kelly 2004).

Trained Rape Crisis Center educators (hereafter “educators”; *n* = 28 educators; all female) delivered a curriculum adapted for high school students. Educators attended a 4-day training delivered by the developer. Research staff, including the developer, reviewed educators’ audio recordings of in-depth bystander training sessions to assess the fidelity of program implementation. Each educator made audio recordings for each training phase (approximately two per educator per year). All audio recorders were reviewed by 2–3 *Green Dot* trained staff who scored each recording for the presenter’s consistency to the *Green Dot* curriculum, use of time in covering topics in the curriculum, and the overall presentation quality. Research staff provided feedback to educators throughout the trial that addressed how well educators connected with the students and consistency of their training with the curriculum. Dr. Dorothy Edwards was responsible for adapting the college-based *Green Dot* curriculum for this high school based setting; Rape Crisis Center staff also provided feedback on curriculum modifications. If an educator was not providing Green Dot training appropriately per the developer’s specifications, remedial training was provided to support the educator’s knowledge and abilities to reach the desired level of proficiency. All educators providing training reached this level or their tasks were reassigned. Educator turnover was an issue as evidenced by 10 of 28 educators providing *Green Dot* training for only one of the four intervention years. Green Dot (intervention) training began Fall 2010 (beginning year 1 [Y1]), with the majority (>50%) of students in intervention schools receiving a 50-min introductory persuasive speech delivered by educators (Phase 1). This school-wide presentation oriented students to their potential role as engaged bystanders and explained how to recognize “red dots” and “green dots.” Green Dot speeches were provided annually to students in the intervention schools. Phase 2 was implemented beginning Spring 2011 (Y2) using the popular opinion leader (POL) strategy, which suggests that training 12–15% of a student body would maximize diffusion of the intervention (Rogers and Cartano 1962). Educators worked with high school staff to identify students as leaders. Leadership qualities were operationalized as students who were respected, followed, or emulated and not necessarily those with academic, athletic, or social leadership skills. These students were invited to participate in intensive (5-h) bystander training. If space permitted, training was also open for other students; we do not have data to estimate the proportion of students in Phase 2 training who were recruited as popular opinion leaders. Both training phases focused on violence victimization, perpetration, and on prosocial behaviors to recognize situations that may lead to violence and to act directly to distract or to delegate tasks to reduce the likelihood of violence). Training focused not only on sexual violence risk but also on sexual harassment, stalking, and partner violence. Both intervention phases were provided at each intervention school however, within the intervention schools not all students received the intervention within a given year. *Green Dot* speeches (Phase 1) were to be provided to all students in intervention year (Y1) and for freshman across Y2-Y4 with a goal of providing *Green Dot* speeches to all students during their high school career. Phase 2 training was designed to target popular opinion leaders, typically 12–15% of a student body. Thus, within a given intervention year and at the student-level approximately 12–15% students would report this in-depth bystander training. At baseline less than 2% of students in intervention schools had heard a speech while in intervention year 1 thru year 4 then training rates were 50.6%, 42.2, 36.4, and 34.9%, respectively. Similarly, for the Phase 2 POL training, at the baseline or pre-intervention year less than 3% of students reported receiving this training which was not delivered until intervention years 1–4. In these years, 9.2, 9.5, 13.6 and 14.5% of students reported receiving bystander POL training in Y1-Y4, respectively.

High schools randomized to the control condition received no additional prevention programming (usual care). Staff monitored new program implementation in control schools over time and confirmed that no bystander programs were implemented. Other violence prevention programming may have been implemented in schools yet our Memorandum of Understanding with schools stipulated that no other *bystander* intervention be implemented during this trial.

## Study Sample

Across the 13 Rape Crisis Centers’ regions, two schools within the 13 regions (*n* = 26) were selected by the Rape Crisis Centers for simple randomization to each condition in this cluster RCT (Coker et al. 2017; Figure 1). Participating high schools signed Memorandums of Understanding indicating willingness to be randomized, to remain in the trial, and to allow data collection for 5 years (Spring 2010–2014). Over the trial period, only two schools dropped out of the study. To maintain ITT analyses, missing school-level data for these two schools were imputed using single imputation (last observation carried forward) because the school-level sample size (*n* = 26) was small for multiple imputation and missingness was due only to school dropout without the option of returning. Upon trial completion, all schools in both conditions had the option to continue implementation or adopt the intervention at no cost to the school. Primary data collection was conducted at schools with all students (Grades 9–12) invited to complete an annual, anonymous survey before intervention implementation (Spring 2010, baseline) and during implementation from 2011 (Y1) through 2014 (Y4) as planned without an early stop. Researchers worked with each school to identify one or 4 days between February and April when the majority of students would be present.

The study protocol was approved by the University of Kentucky IRB (#13–0680-F1V). Passive parental consent methods were used and letters describing the study were mailed to all parents annually. If parents did not want their child to participate, parents were instructed to contact researchers by phone or e-mail so that these students did not receive surveys. At each administration, all students were given the option of refusing to complete the anonymous survey. The 99-item paper and pencil questionnaire was administered by research staff during the school day and typically took 20–45 min to complete. Research staff read elements of assent to all students. Pencils with website and hotline numbers for domestic violence, sexual violence, and depression support agencies were provided to all students.

## Measures

A description of the specific outcome measured at the individual-level and averaged at the school-level over time follows. The outcomes were social norms operationally defined as expression of attitudes which may support or condone violence and measured as dating violence and sexual violence acceptance. Both violence acceptance outcomes were measured using adaptation of existing scales. Researchers with expertise in measuring interpersonal violence and interventions to reduce violence risk were consulted to assist in scale adaptations to reduce the number of items and change the wording to be more relevant for high school students in 2010. Dropped items were endorsed infrequently or those deemed less relevant for the target age group. For both measures the following introduction to the measures was provided “Thinking about your own feelings and beliefs, please indicate how much you personally agree or disagree with each statement. There are no right or wrong responses.” Response options were the same for both sexual and dating violence acceptance measures (Strongly disagree = 0, disagree = 1, agree = 2 and strongly agree = 3); higher scores indicated greater violence acceptance.

### Dating Violence Acceptance

The 5-item Acceptance of General Dating Violence subscale of the 11-item Acceptance of Couple Violence developed by Foshee et al. (1998) for middle school students was used for this RCT (see Table 1). We opted for this subscale because the ‘Green Dot’ approach is gender-neutral in terms of risk of violence and questions were framed to be inclusive of both sexes and of all sexual orientations. All five items (Range 0–15) loaded on one factor (factor loadings ranged from 0.52–0.78); this reduced scale had good internal consistency in the current high school sample (Cronbach’s Alpha = 0.73; mean ± SD = 3.2 ± 2.8, all students; Cronbach’s Alpha =0.743, mean ± SD = 3.7 ± 2.9, males; Cronbach’s Alpha = 0.70, mean ± SD = 2.7 ± 2.6, females).

**Table 1:** RCT Condition and Violence Acceptance, ITT analyses ^1^: All Students and by Sex

School Level Violence Acceptance (IRMA) Scores				
Time All Students	Intervention *N* = 13 Schools	Control *N* = 13 Schools	I-C Difference (95% CI)	Condition x Time F test _DF1,DF2_^*p* value^
				6.95_3,72_^0.0004^
Year 1	5.69 (5.54, 5.83)	5.71 (5.59, 5.83)	−0.03 (−0.22, 0.17)	
Year 2	5.62 (5.41, 5.83)	5.65 (5.46, 5.83)	−0.03 (−0.31, 0.25)	
Year 3	5.25 (5.12, 5.37)	5.64 (5.41, 5.88)	−0.39 (−0.65, −0.13)	
Year 4	4.95 (4.71, 5.20)	5.37 (5.15, 5.58)	−0.41 (−0.75, −0.08)	
Males				4.84 _3,72_^0.004^
Year 1	6.76 (6.58, 6.95)	6.60 (6.45, 6.76)	0.16 (−0.09, 0.41)	
Year 2	6.63 (6.35, 6.92)	6.60 (6.34, 6.86)	0.04 (−0.34, 0.42)	
Year 3	6.23 (6.14, 6.33)	6.60 (6.26, 6.95)	−0.37 (−0.72, −0.02)	
Year 4	6.02 (5.76, 6.28)	6.33 (6.13, 6.54)	−0.31 (−0.63, 0.00)	
Females				6.22_3,72_^0.0008^
Year 1	4.86 (4.69, 5.04)	4.97 (4.85, 5.08)	−0.11 (−0.31, 0.10)	
Year 2	4.84 (4.61, 5.06)	4.86 (4.71, 5.02)	−0.03 (−0.30, 0.24)	
Year 3	4.56 (4.42, 4.70)	4.90 (4.74, 5.07)	−0.34 (−0.56, −0.12)	
Year 4	4.21 (3.97, 4.46)	4.60 (4.39,4.80)	−0.38 (−0.70, −0.06)	
School Level Dating Violence Acceptance (GDVA)^2^ Scores				
Time All Students	Intervention *N* = 13 Schools	Control *N* = 13 Schools	I-C Difference (95% CI)	Condition x Time F test _DF1,DF2_^p value^
				7.07 _3,72_^0.0003^
Year 1	2.73 (2.65, 2.82)	2.75 (2.63, 2.87)	−0.01 (−0.16, 0.14)	
Year 2	2.72 (2.54, 2.90)	2.67 (2.51, 2.82)	0.05 (−0.18, 0.29)	
Year 3	2.45 (2.35, 2.55)	2.74 (2.57, 2.90)	−0.28 (−0.48, −0.08)	
Year 4	2.46 (2.34, 2.59)	2.69 (2.58, 2.80)	−0.23 (−0.40, −0.06)	
Males				5.01_3,72_^0.003^
Year 1	3.41 (3.29, 3.53)	3.37 (3.21, 3.53)	0.04 (−0.15, 0.24)	
Year 2	3.29 (3.05, 3.53)	3.26 (3.06, 3.46)	0.03 (−0.28, 0.34)	
Year 3	3.01 (2.88, 3.14)	3.35 (3.12, 3.58)	−0.34 (−0.61,-0.07)	
Year 4	3.03 (2.92, 3.14)	3.33 (3.21, 3.46)	−0.30 (−0.47, −0.14)	
Females				3.26_3,72_^0.03^
Year 1	2.19 (2.05, 2.33)	2.24 (2.12, 2.36)	−0.05 (−0.24, 0.14)	
Year 2	2.27 (2.08, 2.45)	2.20 (2.06, 2.35)	0.06 (−0.17, 0.30)	
Year 3	2.05 (1.94, 2.15)	2.27 (2.10, 2.43)	−0.22 (−0.42, −0.03)	
Year 4	2.06 (1.89, 2.22)	2.20 (2.07, 2.32)	−0.14 (−0.35, 0.07)	

^1^*.* Illinois Rape Myth Acceptance (IRMA) items: 1) *Girls should have sex with their boyfriend or guy they are dating when he wants. 2) If a guy spends money on a date, the girl should have sex with him in return. 3) Guys should respond to dates’ or girlfriends’ challenges to authority by insulting them or putting them down. 4) If a girl is sexually assaulted while she is drunk, she is to blame for letting things get out of control. 5) Sexual assault charges are often used as a way of getting back at guys. 6) Many girls lead a guy on and then they claim sexual assault. 7) When girls are sexually assaulted, it’s often because the way they said ‘no’ was unclear*

^2^General Dating Violence Acceptance (GDVA) items: 1) *There are times when dating violence between couples is okay. 2) A girlfriend or boyfriend who makes their girlfriend or boyfriend jealous on purpose deserves to be hit. 3) Sometimes violence is the only way to express your feelings. 4) Some couples have to use violence to solve their problems. 5) Violence between couples is a private matter and others should not get in the way or get involved*

### Sexual Violence Acceptance

A modified version of the *Illinois Rape Myth Acceptance Scale (IRMA)* was used to measure sexual violence acceptance. For this RCT, this 20-item IRMA Scale (Payne et al. 1999) was reduced to seven items (see Table 1. Legend for items). This reduced scale (Range 0–21) has had high reliability and construct validity when adapted for college students (Coker et al. 2011), and its internal consistency was good for all students (Cronbach’s Alpha = 0.75; mean ± SD = 6.0 ± 3.4), among males (Cronbach’s Alpha = 0.77; mean ± SD = 7.0 ± 3.6) and females (Cronbach’s Alpha = 0.69; mean ± SD = 5.2 ± 2.9).

Students were also asked about sociodemographic (gender, grade, race/ethnicity, and receiving reduced-price school meals) and violence risk (sexual attraction, current romantic/dating relationship status, seen or heard a parent being physically abused by a partner, and binge drinking in the past month) characteristics.

## Statistical Analysis

Two sets of analyses were conducted and paralleled Hypotheses 1 and 2. For Hypothesis 1, school-level effectiveness analyses were conducted where the intervention exposure was evaluated “as randomized” meaning intervention or control condition. School-level scores were created as cluster means by averaging individual student-level scores within a school for each study year. For Hypothesis 2, student-level efficacy analyses were conducted where the intervention exposure was evaluated ‘as received’ by individual students within the year surveyed. The *Green Dot* POL training groups for these individual student level analyses were defined based on student responses to the training they received. The hierarchical training groups were defined as any Intervention (INT) Phase 2 or POL, INT Phase 1 alone (speeches), or no INT training. Student-level scores for each outcome were created by summing item responses for each scale.

To measure the hypothesized effectiveness of the intervention to reduce sexual and dating violence acceptance scores over time, school-level cluster means were used (Hypothesis 1); student-level responses clustered within schools were used to assess efficacy (Hypothesis 2).

### Intervention ‘as Randomized’ Analyses

The primary effectiveness or intent-to-treat (ITT) analysis was conducted at the school-level comparing conditions as randomized (Intervention [I] or Control [C]) over time). For school-level ITT, annual violence acceptance school averages or school-level scores (*n* = 26) were used as the primary outcome to address the study hypothesis that school-level violence acceptance would have greater declines over time in intervention relative to control schools. To account for the repeated measures at the school-level, using schools as the unit of randomization and analysis, linear mixed models were used to determine effects of condition, time, and condition-time interactions. To test the hypothesis that the intervention resulted in lower violence acceptance over time and by condition, a condition-time interaction was the primary statistical analysis. Therefore, to estimate the longitudinal effect of the intervention over time, linear mixed models included the effects of randomized condition, time (Y1 2010–Y4 2014), baseline values, and the Condition x Time (CxT) interaction on violence acceptance outcomes (PROC GLIMMIX with an AR (1) R matrix and bias-corrected empirical SE estimates) (SAS, version 9.3, 9.4; Kauermann and Carroll 2001). For these ITT analyses, the estimated mean school-level violence acceptance scores (separate models for each acceptance outcome) were presented by condition (and 95% CI) with absolute differences (intervention–control [I–C]; 95% CI) within year, providing an estimate of reduction in violence acceptance attributable to the intervention.

### Intervention ‘as Received’ Analysis

This efficacy analysis was based on student-level data where the intervention exposure was the students self-reported receipt of training within the intervention schools by training phase. Student-level responses were obtained from anonymous surveys; thus, student-level data could not be linked for longitudinal analysis, so analyses were conducted by year. As schools, not students, were randomized, student-level responses were clustered within schools. To account for the clustering of student responses, for each outcome and in each year, linear mixed models (random intercept) were used to compare training groups (PROC GLIMMIX, Unstructured G Matrix); estimates of the mean scores were provided with 95% confidence intervals for each outcome by year. Differences in the mean scores by exposure comparisons were provided within year for the three intervention exposures relative to no training, i.e. students in control schools; receiving any intervention training vs none was also compared. A collapsed exposure was also created by combining both INT Phase 2 and 1 as Any INT relative to the Control condition to provide a simpler comparison of intervention effects.

As training groups were not fully randomized, annual adjusted mean violence acceptance scores (and 95% CI) were provided for the training exposures; covariate-adjustments were made by including gender (male or female), year in school (freshman, sophomore, junior, or senior), race (non-white or white), sexual attraction (exclusively attracted to opposite sex or other) and witnessing intra-parental IPV (yes or no). Potential confounders were identified by considering bivariate associations; simple comparisons of demographic and violence risk characteristics of student receiving intervention training were evaluated using χ^2^ tests. To be consistent with previously performed ITT analyses (Coker et al. 2017), a significance level of 0.01 (2-sided) was used for all statistical tests. For non-randomized analyses, only a subset of pre-planned comparisons was made so corrections for multiple comparisons were not implemented.

A prior manuscript has addressed the effectiveness of the Green Dot intervention to reduce sexual violence perpetration and victimization as the primary outcome for this RCT. The current manuscript addresses the first of 2 secondary outcomes - violence acceptance - hypothesized as the change as the intervention was implemented and result in changes in violence.

## Results

At the school level, two high schools dropped out of the study, one randomized to the control (Y2) and one to the intervention condition (Y4). Within schools, the refusal rates were 0.5% and 13.6% for parents and students, respectively. A total of 73,044 students provided non-missing data across the 5-year trial. Individual students were not followed over time. Instead, the school was the unit of randomization and effectiveness analyses. The same schools were included across the 5-year study period, with the exceptions noted.

As reported elsewhere for this RCT (Coker et al. 2017), over the study period, 104,081 students were present on survey days; and 89,707 completed surveys. These response rates calculations were based on the American Association for Public Opinion Research guidelines (fall) (2015). This rate was 92.6% at baseline and declined to 76.6% in Y4. Response rates were similar in intervention (84.4%) and control (83.4%) schools. Surveys without demographics, violence, or violence acceptance items were excluded (*n* = 10,080) from the analytic sample. Relative to those completing the survey (*n* = 73,044), those with missing data were more likely to have attended Intervention schools, to be male, non-white, and to report physical dating violence or sexual violence perpetration and victimization. Potential mischievous responders were identified (Robinson-Cimpian 2014) and surveys excluded (*n* = 6583) using the following operational definition: those responding as never drinkers reporting symptoms of alcohol abuse, never sexually active responders but pregnant or having children, or those in multiple relationships in the past 12 months yet no relationship in the same time frame for dating violence items. This approach was a conservative approach to limit potential bias introduced by including inaccurate responses. Those excluded due to mischievous responses were more likely than those completing the survey with non-missing or mischievous responses to be male, non-White, and to have experienced or used physical or sexual violence against another high school student. The final analytic sample included 73,044 surveys over 5 years, representing 26 schools. The overall survey completion rate with non-missing date was 81.4% (*n* = 73,044/89,707).

As reported elsewhere, similarities in sociodemographic and violence risk characteristics (school-level averages) between conditions suggested that randomization resulted in comparable schools across conditions (Coker et al. 2017). No differences in year 1 violence acceptance scores were observed by condition or violence acceptance measure for either sexual violence acceptance (IRMA Mean (standard error) in *I* = 5.70 (.04) and C = 5.62 (.04); t test = 1.29; df = 15,897; *p* = .20) nor dating violence acceptance (GDVA in *I* = 2.74 (.03) and in C = 2.70 (.03); t test = 0.97; df = 15,863; p-.33)); t test = 0.97 df = 15,863; *p* = .33).

### School-Level Intervention as Randomized Analysis (ITT)

Greater declines in violence acceptance were observed in I relative to C conditions over time (Y1-Y4) for both mean IRMA scores (see Table 1: CxT, F _3,72_ = 6.95, *p* = .0004 for all students, and for males, F = 4.84, *p* = .004, and females, F = 6.22 *p* = .0008) and GDVA (CxT, F = 7.07, *p* = .0003 for all students; in males, F = 5.01, *p* = .003, and females F = 3.26, *p* = .03). After the intervention was fully implemented (Y3 and Y4), the school-level mean differences by condition (I-C) for IRMA and GDVA scores were significantly (*p* < .001) lower in both Y3 and Y4 among all students. There were differences in intervention effectiveness by sex; IRMA scores were consistently lower in I v C schools in Y3 and Y4 for both sexes, while GDVA scores were consistently lower in I v C for these two full implementation years for males yet only in Y3 for females. At baseline and across the trial, both measures of violence acceptance, IRMA and GDVA, were significantly lower among females than males.

### Student-Level, Intervention as Received Analysis

Our analyses of demographic characteristics of those who did and did not receive intervention training was used to identify confounders for multivariate analyses. Briefly, students in intervention schools who did not receive intervention training were more likely to be male, seniors, non-white, receiving free or reduced price meals (proxy for family income), not exclusively attracted to the opposite sex, and had witnessed parental intimate partner violence (IPV). Subsequent models were adjusted for sex, grade in school, race, sexual attraction and parental IPV as those demographic factors not collinear with other demographics. Receipt of a free or reduced-price meal was not included as a covariate because it was highly collinear with race and other covariates.

The number and proportion of students self-reporting receipt of intervention training by Phase and Condition were calculated over time and by sex. More than half of students in intervention schools received either Phase 1 or 2 training between Y1 and Y4 (*n* = 16,492; 60.6% - 50.1%); 13,129 recalled hearing a speech (52.4–37.0% in Y1-Y4). Phase 2 intensive training was delivered in groups (mean group size, 32 students; range, 17–60) held during school hours, with at least two trainings per academic year per school. A total of 3363 students received bystander training (Phase 2: 8.3% in Y1–13.2% in Y4). In Table 2, measures of intervention efficacy were reported as the differences in IRMA and GDVA adjusted means by training within intervention schools relative to means for students in control schools. Mean differences were presented for all students and by sex.

**Table 2:** Student-Level Data, Evaluation Based on Student Reports of Training Received by Intervention and Control Conditions

Time	Difference in Adjusted Mean* IRMA Scores (95% CI for mean) for Students in Intervention [I] Schools by Training Received – Scores for Students in Control [C] Schools			
All Students	[I] Any GD training – [C]	[I] Phase 2 – [C]	[I] Phase 1 only - [C]	[I] No GD Training – [C]
Year 1	−0.24 (−0.55, 0.07)	0.10 (−0.28, 0.48)	−0.29 (−0.58, −0.00)	0.35 (0.06, 0.65)
Year 2	−0.27 (−0.60, 0.05)	−0.13 (−0.49, 0.24)	−0.31 (−0.62, −0.01)	0.33 (0.03, 0.64)
Year 3	−0.54 (−0.86, −0.23)	−0.61 (−0.97, −0.26)	−0.52 (−0.82, −0.22)	−0.02 (−0.32, 0.27)
Year 4	−0.49 (−0.89, −0.09)	−0.46 (−0.88, −0.03)	−0.50 (−0.89, −0.12)	−0.11 (−0.49, 0.27)
Males				
Year 1	−0.20 (−0.57, 0.16)	0.32 (−0.21, 0.85)	−0.29 (−0.64, 0.05)	0.74 (0.39, 1.09)
Year 2	−0.30 (−0.76, 0.16)	0.03 (−0.53, 0.59)	−0.39 (−0.83, 0.05)	0.54 (0.11, 0.96)
Year 3	−0.69 (−1.09, −0.29)	−0.69 (−1.23, −0.16)	−0.69 (−1.08,-0.29)	0.20 (−0.17, 0.56)
Year 4	−0.51 (−0.95,-0.07)	−0.13 (−0.67, 0.41)	−0.64 (−1.07, −0.22)	0.06 (−0.34, 0.45)
Females				
Year 1	−0.27 (−0.59, 0.05)	−0.06 (−0.47, 0.35)	−0.30 (−0.6,0 0.00)	−0.01 (−0.32, 0.30)
Year 2	−0.27 (−0.57, 0.03)	−0.26 (−0.63, 0.11)	−0.27 (−0.56, 0.01)	0.14 (−0.16, 0.43)
Year 3	−0.45 (−0.77, −0.14)	−0.57 (−0.94,-0.20)	−0.41 (−0.72, −0.10)	−0.20 (−0.51, 0.10)
Year 4	−0.51 (−0.95, −0.07)	−0.67 (−1.13,-0.20)	−0.44 (−0.87, −0.01)	−0.27 (−0.69, 0.15)
Time	Difference in Mean* GDVA Scores (95% CI for mean) for Students in Intervention [I] Schools by Training Received – Scores for Students in Control [C] Schools			
All Students	[I] Any GD training – [C]	[I] Phase 2 – [C]	[I] Phase 1 only - [C]	[I] No GD Training – [C]
Year 1	−0.23 (−0.41, −0.04)	0.06 (−0.20, 0.32)	−0.27 (−0.45, −0.10)	0.24 (0.06, 0.43)
Year 2	−0.18 (−0.42, 0.07)	−0.05 (−0.33, 0.23)	−0.21 (−0.44, 0.02)	0.26 (0.03, 0.49)
Year 3	−0.45 (−0.65, −0.25)	−0.61 (−0.86, −0.36)	−0.39 (−0.59, −0.19)	−0.04 (−0.23, 0.16)
Year 4	−0.36 (−0.56, −0.16)	−0.36 (−0.59, −0.12)	−0.36 (−0.55, −0.16)	−0.04 (−0.23, 0.14)
Males				
Year 1	−0.21 (−0.47, 0.04)	0.16 (−0.25, 0.56)	−0.28 (−0.52, −0.03)	0.42 (0.17, 0.67)
Year 2	−0.23 (−0.57, 0.12)	−0.01 (−0.44, 0.42)	−0.29 (−0.62, 0.04)	0.32 (0.00, 0.64)
Year 3	−0.60 (−0.90, −0.31)	−0.72 (−1.12, −0.32)	−0.57 (−0.86, −0.28)	0.06 (−0.21, 0.32)
Year 4	−0.48 (−0.71, −0.25)	−0.36 (−0.71, −0.01)	−0.52 (−0.75,-0.29)	−0.07 (−0.27, 0.14)
Females				
Year 1	−0.23 (−0.41, −0.04)	0.01 (−0.29, 0.30)	−0.26 (−0.44, −0.08)	0.10 (−0.09, 0.29)
Year 2	−0.14 (−0.36, 0.08)	−0.09 (−0.37, 0.20)	−0.16 (−0.37, 0.05)	0.20 (−0.01, 0.42)
Year 3	−0.34 (−0.54, −0.13)	−0.54 (−0.80, −0.27)	−0.26 (−0.46, −0.06)	−0.11 (−0.31, 0.09)
Year 4	−0.28 (−0.51, −0.05)	−0.36 (−0.63, −0.08)	−0.25 (−0.48, −0.02)	−0.03 (−0.25, 0.19)

*All models were adjusted for gender, grade, race, sexuality and witnessing IPV. Gender was dropped for sex specific models. Mischievous respondents were excluded from all analyses

### Any Intervention Training Relative to Control Condition (I-C)

When compared with students attending schools randomized to the control condition, both IRMA and GDVA scores were significantly and consistently lower for students receiving any intervention training (Phase 1 or 2) beginning in Y3 and continuing in Y4. This pattern was observed for both sexes.

### Phase Relative to Control Condition (I-C)

Phase 2 trained students had consistently lower IRMA scores than controls in both Y3 and Y4 among all students and females, yet only in Y3 was this I-C significantly different among males. In Y3 and Y4, Phase 2 training was associated with a significant reduction in GDVA scores for all students and by sex relative to controls. In Y3 and Y4, Phase 1 training was associated with significant reductions in both IRMA and GDVA scores for all students and by sex relative to controls. In Y1, Y2, Y3, and Y4, Phase 1 trained students had consistently lower IRMA and GDVA scores than in controls among all students.

Notably, IRMA and GDVA scores were *higher* in Y1 and Y2 among all students (specifically males), attending intervention school yet not receiving training ([I] no GD Training) relative to controls. With full intervention implementation by Y3 and Y4, these differences in IRMA and GDVA scores were no longer significant. This reduction in violence acceptance scores among those not trained yet attending intervention schools suggested that intervention training at the school level may be diffused to non-trained students and, thereby, changed their attitudes regarding violence acceptance.

## Discussion

Results from this 5-year RCT indicated that the bystander program Green Dot, adapted for high school students and delivered by trained Rape Crisis Center educators, was both effective (as randomized) and efficacious (as received) in reducing sexual and dating violence acceptance scores. Consistent with the effectiveness hypothesis stated as Hypothesis 1, both IRMA and GDVA mean scores declined more rapidly over time in the intervention relative to control schools as evidenced by significant condition x time interactions for all students and by sex.

Intervention as received analyses at the student level and based on the students’ self-report of training received provided further evidence to support Hypothesis 2. Relative to the control condition, students who received intervention training (Phase 1 or 2) had lower IRMA and GDVA mean scores in year 3 and 4 when the intervention was fully implemented. This pattern held for GDVA among both sexes and for IRMA for females and in year 3 for males.

Bystander programs were hypothesized to reduce violence acceptance for those trained and over time to change violence acceptance and violence rates at the school level. Data from this 5-year trial supported this hypothesized patterning and indicated that sufficient time was required to see the ultimate effect of the bystander training on violent acceptance and behaviors. The observed time delay between intervention implementation and reductions in violent acceptance scores was anticipated because the intervention was hypothesized to reduce violence acceptance by first changing individual-level social norms supporting violence among the subset of trained individuals (measured by student-level efficacy analysis) and ultimately changing violence acceptance at the school-level, as the intervention was implemented (Y1-Y4) with full implementation by Y3-Y4. Finding significant reductions in Phase 1 trained intervention students for IRMA (in Y1-Y4) and GDVA (in Y1, Y3 and Y4) relative to those in control schools suggested that motivational speeches alone affected violence acceptance particularly when sufficient numbers of students were trained (here approximately 50% in year in Y1). Further, with full implementation (Phase 1 and 2), individual-level changes in violence acceptance appeared to impact violence acceptance among those in the intervention schools who did not receive training. This finding suggested diffusion of the training intervention at the school level. This intervention diffusion had direct implications for changing the social norms and culture within a school.

## Research Implications

The identification of Green Dot as an effective intervention for reducing student and school-level violence acceptance in combination with recently published findings that Green Dot appeared to reduce violence rates (Coker et al. 2017) provides additional evidence for bystander programming effectiveness and efficacy. The current research indicates that Green Dot changes *both* sexual violence (IRMA) and dating violence (GDVA) acceptance scores. This report provides support for the mechanism by which the intervention may change violence rates by changing violence acceptance. The additional contribution of the current analyses was the evidence that changes in violence acceptance in the intervention relative to control schools (effectiveness) and among those receiving training (efficacy) may explain the observed reductions in violence rates over time.

This study is the first RCT of a bystander intervention focusing on violence prevention and in this analysis for violence acceptance as a secondary outcome, implemented with both sexes in a high school setting. This intervention was unique in its use of a popular opinion leader model (Phase 2) for recruitment and training is a particularly efficient method to diffuse prosocial, non-violent norms through students’ peer networks. In this current analysis both dating violence and sexual violence acceptance scores were lower among those receiving either of the two intervention Phases relative to no training. This finding contrasts with those from a college sample where only a reduction in sexual violence acceptance was observed for those receiving training (Coker et al. 2011). It is relevant to note that *Green Dot’s* training focus is primarily sexual violence. The larger sample size for this high school based study may explain finding reductions in both sexual and dating violence acceptance in the high school yet not the small college-based study.

Modest sex differences in the effectiveness and efficacy of training were observed. Both IRMA and GDVA means were significantly lower among females than males at baseline and over time. At the school-level effectiveness analysis, the intervention was associated with reductions in both IRMA and GDVA in males and females (Table 1). For student-level efficacy analyses, both GDVA and IRMA means were significantly lower in Y3 and Y4 among those receiving training (Phase 1 or 2) relative to control students for males and females. The reduction appeared to be greater among males than females. Given the already lower violence acceptance scores among females relative to males, the impact (need) of the intervention may be greater among males. More females received training (particularly Phase 1) than males. Specific recruitment of popular opinion leaders, used in Phase 2 training, was a particularly effective approach to increase the number of males receiving intervention training.

A unique contribution of the current analysis is our ability to measure the impact of the intervention at the school and student levels. With both the school and student level analyses we can determine both effectiveness and efficacy of the intervention to change violence acceptance and eventually reduce violence perpetration and victimization (Coker et al. 2017). Our school-level effectiveness analysis suggest that *Green Dot* reduces two measures of violence acceptance even when half the students are not directly receiving this training. The effect of the training appear to be diffused to non-trained students via trained students. This training diffusion model can be quite efficient. This school-level effectiveness analysis provides a ‘real world’ indicator of intervention’s impact given that school-based interventions are difficult to implement with fidelity, over a sustained and long period, and to the majority of students. The student-level efficacy analysis suggests the effect of training actually received on concurrent violence acceptance scores. A limitation of this analysis is that lack of pre and post intervention assessment of violence acceptance scores because the student surveys were anonymous. However, it is likely that the impact of training on violence acceptance is likely to be relatively rapid. The analyses at the school level allowed a more macro or *community change effect* of the intervention on this same set of violence acceptance measures. Prior violence prevention evaluation research was conducted at the school level; several using this approach have focused on bullying prevention or interventions (Polanin et al. 2012; Whitted and Dupper 2005). Several evaluations with dating violence as the primary outcome used a cluster design with school-level clustering and analyses at the individual level, where students were followed over time (Foshee et al. 1998; Taylor et al. 2013; Tharp et al. 2011). While “Coaching Boys into Men” (Miller et al. 2012) and the one-year follow-up (Miller et al. 2013) focused on dating violence perpetration as its primary outcome, this research was perhaps most comparable to the current study in its similar settings and comparable intervention approach that included bystander elements. In contrast with Miller et al. 2012, 2013, the current study included both young men and women in the intervention training and evaluation.

Few studies have evaluated intervention efficacy on changes in violence acceptance over time, specifically, at the school level. In the Men’s Project Evaluation, Gidycz et al. (2011) reported no change in rape myth acceptance over 7 months’ time among men. Foubert (2000) used the “The Men’s Program” and found a decrease in rape myth acceptance over time among fraternity members. When evaluating Safe Dates Foshee et al. (1998) observed significant changes in social norms over time by condition as hypothesized to be consistent with intervention implementation and effectiveness; changes in social norms patterns differed by those experiencing and using violence. Using the same Green Dot high school evaluation data (https://clinicaltrials.gov/show/NCT01878097), both sexual and dating violence acceptance were strong mediators of the effect of the Green Dot intervention on reducing sexual violence outcomes over time and in Y3 and Y4 (Heather M Bush, PhD, personal communication).

## Clinical and Policy Implications

The bystander intervention Green Dot was found to work in terms of the pathway of the intervention. According to bystander theory the intervention should reduce violence acceptance and increase bystander behaviors resulting in a reduction of violence over time (Coker et al. 2017). While Green Dot has been found to reduce sexual and dating violence perpetration and victimization among high school students (Coker et al. 2017) additional research and policies supporting bystander interventions are needed to maintain a reduction in violence over time.

## Limitations

As noted elsewhere (Coker et al. 2017), Green Dot was implemented in Kentucky high schools with thorough intervention training and fidelity throughout the implementation, which may have contributed to programmatic success. Results of this trial may not generalize to other settings if implemented with different educator training or fidelity.

Although an experimental study design was used in this trial, all outcomes were self-reported. While self-reports are the *only* data source of students’ attitudes toward violence (sexual and dating violence acceptance), this study’s validity remains dependent on the accuracy of students’ self-reports. Lack of blinding of intervention status may have led to a social desirability bias in responses to the violence acceptance measure, such that students in intervention schools may have under-reported violence acceptance because they knew their school had a violence prevention program. To address this potential threat study to validity, data collection was anonymous; there was no way to link individual students by training and over time. Measures of violence acceptance were not labeled or framed as norms or attitudes but that of opinions with no right or wrong answers using established scales (IRMA and GDVA). Finally, to address students not taking time and care to provide accurate responses, and given the study sample size, analyses were conducted to exclude mischievous responders instead of adjusting for correlates of mischievous respondents as proposed by Robinson-Cimpian (2014).

Individuals were not tracked over time as surveys were anonymous. However, the study was designed to measure intervention associated change in violence acceptance at both the student-level efficacy analysis and the school-level effectiveness analysis. The latter was consistent with the Green Dot model for intervention diffusion where training impacts individuals, their social networks and ultimately violence acceptance changes at the school level. The addition of intervention efficacy analyses provided a more comprehensive indicator of how Phase 1 and 2 training resulted in violence acceptance within intervention schools and relative to control school over time. Future research to measure bystander effectiveness in changing violence acceptance and violence rates for trained individual, through their social network, within their schools, and into their communities would add to the existing literature.

## Conclusions

Implementation of a bystander intervention to reduce violence acceptance in Kentucky high schools decreased both sexual and dating violence acceptance over time with program implementation. The impact of intervention training on reducing violence acceptance was greatest among those receiving training (efficacy analyses); yet, training had an effect on reducing violence acceptance at the school level and over time with intervention implementation. Further studies are needed to assess bystander intervention effectiveness and efficacy in other settings. These findings are among the first to identify an effective bystander intervention to reduce sexual and dating violence acceptance.
